# Adverse outcomes associated with opioid prescription for acute low back pain: a systematic review protocol

**DOI:** 10.1186/s13643-017-0556-x

**Published:** 2017-08-14

**Authors:** Natalia Mouravska, Laura Zielinski, Meha Bhatt, Nitika Sanger, Monica Bawor, Brittany Dennis, Laura Banfield, James MacKillop, James Paul, Andrew Worster, Philip Laplante, Lehana Thabane, Zainab Samaan

**Affiliations:** 1 Juravinski Hospital, Hamilton Health Sciences, 711 Concession Street, Hamilton, ON L8V 1C1 Canada; 2 Mood Disorders Research Unit, St. Joseph’s Healthcare Hamilton, 100 West 5th Street, Hamilton, ON L8N 3K7 Canada; 3 MiNDS Neuroscience Graduate Program, McMaster University, 1280 Main Street West, Hamilton, ON L8S 4L8 Canada; 4 Peter Boris Centre for Addictions Research, St. Joseph’s Healthcare Hamilton, 100 West 5th Street, Hamilton, ON L8N 3K7 Canada; 5 Population Genomics Program, Chanchlani Research Centre, McMaster University, 1280 Main Street West, Hamilton, ON L8S 4L8 Canada; 6 Health Research Methodology Graduate Program, McMaster University, 1280 Main Street West, Hamilton, ON L8S 4L8 Canada; 7 Department of Psychiatry and Behavioural Neurosciences, McMaster University, 1280 Main Street West, Hamilton, ON L8S 4L8 Canada; 8 St. George’s University of London, Cranmer Terrace, London, SW17 0RE UK; 9 Health Science Library, McMaster University, 1280 Main Street West, Hamilton, ON L8S 4L8 Canada; 10 Department of Anaesthesia, McMaster University, 1280 Main Street West, Hamilton, ON L8S 4L8 Canada; 11 Department of Medicine, Hamilton General Hospital, 237 Barton St East, Hamilton, ON L8L 2X2 Canada; 12 Department of Health Research Methods, Evidence, and Impact, McMaster University, 1280 Main Street West, Hamilton, ON L8S 4L8 Canada; 13 Biostatistics Unit, Research Institute at St Joes, St. Joseph’s Healthcare Hamilton, 50 Charlton Avenue E, Hamilton, ON L8N 4A6 Canada; 14 Mood Disorders Program, St. Joseph’s Healthcare Hamilton, 100 West 5th St, Hamilton, ON L8N 3K7 Canada

**Keywords:** Opioid use, Acute lower back pain, Systematic review, Prescription opioid, Protocol

## Abstract

**Background:**

Acute low back pain (ALBP) is the top cause of global disability, demonstrating a significant impact on individuals and society and demanding the need for appropriate management. There is a trend towards an increasing number of opioid prescriptions for ALBP despite the lack of investigation for its various short- and long-term outcomes. The objective of this review is to examine adverse outcomes associated with opioid use for ALBP.

**Methods/design:**

Using a search strategy, the search will be conducted using the following electronic databases: PubMed/MEDLINE, EMBASE, PsycINFO, Cumulative Index to Nursing and Allied Health Literature (CINAHL), Web of Science, Cochrane Library, the National Institutes for Health Clinical Trials Registry and the World Health Organization International Clinical Trials Registry Platform (WHO ICTRP). We will include randomized clinical trials and observational studies investigating the impact of opioid use in ALBP in the adult population. All phases of screening, data extraction and assessment of methodological quality will be performed by two independent reviewers. We will perform quality and risk of bias assessment for the included articles and compare high and low risk of bias with a sensitivity analysis. We will conduct random- and fixed-effects meta-analyses with heterogeneity calculated using the *I* ^*2*^ statistic and evaluate publication bias.

**Discussion:**

There are current guidelines published to alert clinicians in prescribing opioids for ALBP due to its likelihood of misuse, yet there is little change in prescribing patterns. To date, there is an absence of systematic information about the outcomes of prescription opioid in patients with ALBP. We will address this gap by providing evidence that will be useful for clinical practice.

**Systematic review registration:**

PROSPERO CRD42016033090

**Electronic supplementary material:**

The online version of this article (doi:10.1186/s13643-017-0556-x) contains supplementary material, which is available to authorized users.

## Background

Low back pain is a common complaint for adult patients presenting to primary care physicians [1, 2]. It can be debilitating to patients resulting in lost productivity leading to high economic burden due to direct and indirect costs [3]. Acute low back pain (ALBP), defined as pain lasting a minimum of 1 day [4], is ranked as the leading cause of global disability [4]. ALBP is characterized by pain or discomfort between the costal margin and inferior gluteal folds, lasting for less than 12 weeks [5, 6]. This condition has a significant impact on the diagnosed individuals and has a need for appropriate management. Although a large proportion of ALBP patients recover within 2 weeks, recurrent pain is experienced by up to 70% of ALBP patients within 12 months of onset [7, 8].

About 85% of acute low back pain is nonspecific and therefore cannot be attributed to a definite cause [9]. However, possible causes of acute low back pain are many and may include inflammatory, malignancy, infective, and traumatic origins among others which need to be assessed based on the patient’s history and physical examination [9].

Current guidelines by the American College of Physicians, American Pain Society and the European guidelines for the management of acute non-specific low back pain in primary care recommend the use of non-opioid therapies, such as non-steroidal anti-inflammatory drugs (NSAIDs), as the first line of treatment of ALBP [5, 10, 11]. Guidelines propose that opioid analgesics be used for ALBP only in severe cases, specifically when other forms of treatment are ineffective [5, 10, 11]. However, there is an increasing number of opioid prescriptions for ALBP despite the lack of consistent evidence to support its effectiveness [12]. In addition, studies have shown that work loss associated with back pain was 11 to 14 times more likely for patients who received opioids compared to those without opioid treatment [13]. There is no current knowledge synthesis on the outcomes of opioid use for ALBP to address the potential for substance misuse, social problems and physical adversities. A recent study reported more than half of the women and a third of the men with opioid use disorder were first exposed to opioids through a physician prescription for opioids [14]. Similarly, 50% of the patients attending treatment for opioid use disorder started using opioids following pain, injury, surgery or a dental procedure [15]. Although previous systematic reviews have examined the effectiveness of opioids in relieving pain in the short term [16], there is a gap in the literature summarizing the long-term incidence of misuse, abuse or dependence following prescription opioids for acute pain.

Misuse of prescription opioids has reached alarming proportions in Canada and continues to increase at the global level [17]. In 2013, the Canadian Centre of Substance Abuse (CCSA) released a strategy for the prevention of prescription drug abuse and dependence as a response to the crisis in Canada [18]. The strategy outlines prevention, education and treatment recommendations that emphasize the development of evidence-informed policies to avoid harm from prescription drugs as well as educational programming for clinicians surrounding addictions and improved prescribing practices [18]. Despite these recommendations, the use of opioids continues to be a challenge. In Ontario, deaths related to prescription opioids have doubled since 1991 [19], and recent data show 10 accidental deaths related to prescription opioids occur every week [20]. Non-medical opioid use, which includes the use of over the counter or prescription opioids outside what the physician has prescribed, results in detrimental consequences for individuals and the society. These include increased risk of infection, criminality, health care costs, addiction and mortality [21].

In the absence of effective prevention and treatment strategies, the costs and harms associated with opioid use are expected to rise. With back pain being the top cause of global disability, opioids are commonly used for back pain and with more than half of ALBP patients experiencing recurrent pain, it is necessary to understand the long-term outcomes of opioid analgesic use to better aid physicians in treating ALBP.

### Objectives

The proposed review aims to examine the incidence of opioid misuse, abuse or dependence, physical adverse events (i.e. withdrawal symptoms, emergency room visits, opioid intoxication, hospitalizations due to opioid use), social adversity (i.e. criminal activity, unemployment, marital discord) and mortality in adult ALBP patients treated with opioid analgesics. Specifically, our aims are as follows:

1. Conducting a systematic review and meta-analysis to assess adverse outcomes of prescription opioid use for acute lower back pain which includes determining the associated risk of continued opioid use and adverse social and health outcomes in ALBP patients treated with opioids
2. Identifying the characteristics of ALBP sufferers who develop side effects associated with opioids prescribing/use

## Methods

### Protocol and registration

This protocol is presented in accordance with the Preferred Reporting Items for Systematic Review and Meta-Analysis Protocols (PRISMA-P) guidelines [22]. The PRISMA-P checklist is included as an additional file [see Additional file 1]. This protocol is registered with PROSPERO no. CRD42016033090.

### Data source and search strategy

An experienced librarian (LB) will be consulted when devising and implementing the search strategy. A broad search strategy will be employed to include titles, abstracts and keyword fields as shown in Table 1. No language constraints will be included in the search strategy. The searches will be limited to adult human studies. We will search the following databases: PubMed/MEDLINE, EMBASE, PsycINFO, Cumulative Index to Nursing and Allied Health Literature (CINAHL), Web of Science, the National Institutes for Health Clinical Trials Registry and the World Health Organization International Clinical Trials Registry Platform (WHO ICTRP). Articles will be identified using a comprehensive search strategy modified for each database. The search strategy will include all relevant search terms related to ALBP and prescription opioids and their medical subject headings. Databases will be searched from inception to present. In addition, we will manually search through the reference lists of articles that pass the initial abstract screening for any relevant articles the search strategy may not have captured. We will also search key journals to identify relevant articles. Sources of grey literature including dissertations and theses, clinical guidelines and reports from regulatory agencies will be searched. Reference lists of relevant systematic reviews and all included studies will be checked to identify additional articles.

**Table 1:** Search strategy for extraction of relevant studies

Database	Search strategy
MEDLINE	1. exp acute pain/ 2. exp. low back pain/ 3. exp. analgesics, opioid/ 4. exp. morphine/ 5. exp. codeine/ 6. exp. fentanyl/ 7. exp. tramadol/ 8. exp. meptazinol/ 9. exp. pentazocine/ 10. exp. methadone 11. exp. buprenorphine/ 12. oxycodone.mp. 13. dipipanone.mp. 14. remifentanil.mp. 15. papaveretum.mp. 16. pethidine.mp. 17. tapentadol.mp. 18. 1 or 2 19. 3 or 4 or 5 or 6 or 7 or 8 or 9 or 10 or 11 or 12 or 13 or 14 or 15 or 16 or 17 20. 18 and 19 21. Limit 20 to humans
EMBASE	
PsychINFO	
CINAHL	
Web of Science	
Cochrane Library	
Cochrane Clinical Trials Registry	
National Institutes for Health Clinical Trials Registry	
World Health Organization International Clinical Trials Registry Platform	

### Inclusion and exclusion criteria

We will include both randomized controlled trials (RCTs) and observational studies in the review. We will include pilot or feasibility studies that are powered to provide conclusions on the intervention and look at the desired outcomes. Other trial designs, namely cross-over and cluster RCTs, will be included in this review although it is likely that such designs may be rare in studies of opioid analgesics for ALBP. Trials are expected to test the effectiveness of opioids for pain relief in patients with ALBP as well as report adverse events including misuse, overdose and mortality. Observational studies (including cohort and cross-sectional studies with adjusted analyses) examining outcomes of prescription opioid use for ALBP will be included in this review. We will include studies investigating the use of prescription opioids (morphine, diamorphine, fentanyl, alfentanil, remifentanil, methadone, oxycodone, pethidine, tapentadol, tramadol, codeine, dihydrocodeine, meptazinol) for ALBP and reporting effectiveness, side effects, duration of use, post-intervention follow-up, incident misuse, social adversity (for example, marital/relationship problems and employment problems) and mortality. The comparators include placebo, non-opioid analgesics, psychotherapy or alternative/complementary therapies such as acupuncture, yoga, physiotherapy, hydrotherapy and heat application. We will be including any studies that are looking at a co-intervention of opioid substitution therapy (OST) if they report on opioid-related outcomes.

Participants must be aged 18 years or older. No restrictions will be placed on upper age limit. No restrictions will be placed on sex, ethnic background or participants’ main language. Studies will be included in this review if the primary diagnosis of the study participants is ALBP as defined by reporting low back pain of ≤12 weeks without a clear and specific attributable cause (trauma, osteoporotic fractures, infections, malignancy and mechanical derangement) [6]. Patients with comorbid substance use disorders will be included, as it is likely that these patients are susceptible for opioid misuse. Studies in any setting will be included such as primary care, hospitalized or community based.

### Outcome measures

The primary outcome is adverse events including incidence of misuse including opioid use disorder, physical adverse events of withdrawal symptoms, opioid intoxication, emergency room visits, hospitalizations, social adversity of criminal activity, unemployment, marital discord and mortality.

Outcomes will be assessed at the end of the treatment period for all included studies. As it is expected that the treatment periods will vary among studies, we will use a predetermined time point of treatment completion of 3 months. We will group the studies based on durations of less than 3 months and 3 months and more. The 3 months were selected based on the longest period expected for acute pain, as explained previously. We will also consider the outcomes at follow-up if available. We would like to investigate both short- and long-term outcomes, and there is little in the literature to help define these lengths of outcomes in ALBP as study timelines vary. We had to make a pre-specified definition. Follow-up durations will be grouped into less than 6 months or 6 months or more.

### Selection of studies

Two reviewers will independently screen title and abstracts for inclusion in this review. Two reviewers will perform full-text review and data extraction independently. Disagreements between reviewers will be resolved by discussion to consensus or by consulting a third party if it remains unresolved. We will assess agreement between reviewers and report the kappa statistic within our results. A Preferred Reporting Items for Systematic Reviews and Meta-Analyses (PRISMA) [22] flow diagram outlining all phases of screening and reasons for exclusions will be included.

### Data extraction and management

All of the studies and references will be managed and organized through an online software program Refworks. Full-text data extraction forms will be constructed to include the following information: author; year of study; country; number of participants; mean age and sex ratio; study methodology (RCT, cohort, cross-sectional); definition of ALBP; type of prescription opioid (morphine, codeine, etc.); dose and duration of treatment; outcomes assessed in the study; statistical measurements and statistical results (*p* values, confidence intervals, effect measures, etc.). The data extraction form will be pilot tested by two independent reviewers to determine feasibility in this review. The data extraction form is included as an additional file [see Additional file 2].

### Quality assessment and risk of bias

Two reviewers will independently assess the methodological quality of eligible studies for this review.

A modified version Newcastle-Ottawa Scale [23] that has been previously used in cross-sectional studies will be used to assess the risk of bias for the nonrandomized studies. Each study will be judged on eight items, categorized into three groups: the selection of the study groups, the comparability of the groups and the ascertainment of the exposure or outcome of interest for case-control or cohort studies. A study can be awarded a maximum of one star for each numbered item within the selection and exposure categories. A maximum of two stars can be given for comparability such that the highest quality studies are awarded up to nine stars. To assess the risk of bias for randomized control studies, Cochrane Risk of Bias tool will be used to closely examine selection bias, performance bias, detection bias, attribution bias, reporting bias, and other sources of bias.

The strength of evidence will be examined using the Grading of Recommendations Assessment, Development and Evaluation (GRADE), which scores according to risk of bias, publication bias, consistency, directness and precision [24]. Treatment comparisons will be given one of the four GRADE scores reflecting the quality of the evidence—high-, moderate-, low-, or very low-quality [24] evidence which will be summarized in a table.

### Data synthesis and statistical analysis

Results from this systematic review will be summarized both qualitatively and quantitatively where possible. We will report on prescription patterns in observational studies, doses and types of opioids selected, duration of treatment and whether any specific guidelines were followed. We will also describe the characteristics of patients who experience adverse outcomes following prescription opioids. We will analyze dichotomized data as odds ratio (OR) with 95% confidence intervals for each outcome. For continuous data, we will analyze the data as mean difference (MD) or standardized mean difference (SMD). All direct estimates will be pooled separately based on study design (randomized vs. non-randomized). While some studies suggest the differences in treatment estimates obtained from well-designed observational research do not differ greatly from RCTs on the same variables [25, 26], pooling data from observational studies and RCTs is highly cautioned against [27–29]. This separation stems largely from the inherent differences between RCTs and observational designs, whereby non-randomized designs face high susceptibility to selection bias [28]. Considering the expected degree of heterogeneity across studies, we will use the random-effects model which accounts for variability within and between studies and yields a more conservative estimate than the fixed-effects model [30]. We aim to pool the results (when possible) of randomized and non-randomized studies separately. We will use adjusted analyses from observational studies to account for confounding. If the randomized and non-randomized studies differ in direction, we will conduct post hoc subgroup analyses to determine if there are any confounding factors in the observational studies that are causing this effect. Heterogeneity will be assessed using the *I* ^2^ statistic. It is advised not to impose cut-off values of heterogeneity because the importance of heterogeneity depends on a multitude of factors. However, Cochrane suggests a value < 40% which may not represent a notable amount of heterogeneity [31]. Thus, possible sources of clinical heterogeneity will be examined given an *I* ^2^ statistic > 40%. A meta-analysis will be completed where possible using STATA Version 13 and Review Manager 5.2. The results of the pooled analysis will be presented in forest plots. We will present Egger’s plot to assess publication bias among the pooled studies.

### Dealing with missing data

We will contact study authors in order to obtain missing numerical outcome data where possible. We will document all correspondence with authors. If we still have missing data, we will use imputation strategies following Ebrahim et al.’s methods [32] for continuous outcomes and Akl et al. for dichotomous outcomes [33].

### Subgroup analysis

If substantial heterogeneity is present, subgroup analyses to explain clinical heterogeneity will be conducted based on study age group, sex, type of opioid and opioid dose (converted to morphine milligram equivalent), duration of treatment and follow-up and type of population (comorbid vs. no disorders). In regard to opioid dose, we will have the low to moderate dosage cut-off at 90 mg morphine equivalents and below and the high dosage will be anything above 90 mg morphine equivalents according to the 2017 Canadian Guideline for Opioids for Chronic Non-Cancer Pain [34]. In addition to investigating heterogeneity, we will hypothesize a priori sources of heterogeneity and conduct subgroup analyses based on sex as we have shown in previous studies [14] that opioid use disorder can vary by sex and therefore sex is an important factor in determining outcomes following opioid use. In addition, pain tolerance and treatment outcomes are also varied by sex [35] making this subgroup analysis essential for this study.

### Sensitivity analysis

Sensitivity analyses will be conducted to assess the robustness of the results by analyzing only complete sets of data excluding imputed data. Also, in the case of having a small number of studies in the meta-analysis, the commonly used variance-inverse method may not be the most appropriate method in this instance and therefore we will perform sensitivity analysis using the small-sample-adjusted methods of Knapp-Hartung, likelihood profile and Bayesian hierarchical approach [36] to assess the robustness of the results [37].

### Presenting and reporting of results

**Fig. 1:**
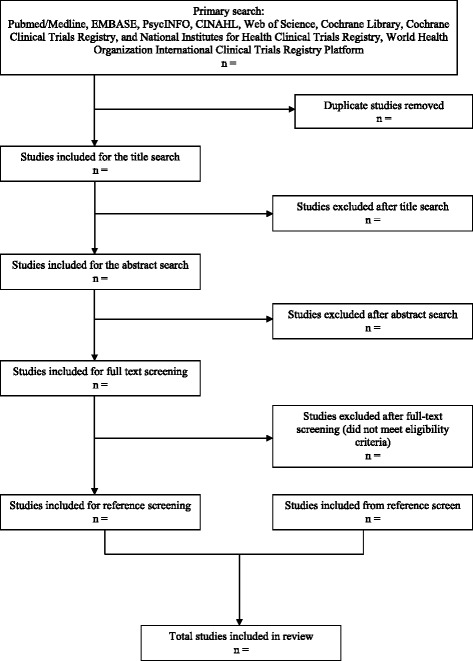
Flow diagram of included studies

The full review will follow the Meta-analysis of Observational Studies in Epidemiology (MOOSE) and PRISMA reporting guidelines [38]. A flow chart will display the phases of screening and selection of articles, with reasons for exclusion (Fig. 1). If meta-analysis is possible, we will present results in a forest plot. We will also include detailed table of included studies following the final screening stage, in accordance with the MOOSE guidelines [39].

## Discussion

Current guidelines for acute pain management recommend cautious prescribing of opioid analgesics and thorough assessment of individuals with ALBP in primary care settings [40]. However, opioids continue to be prescribed for ALBP by primary care physicians and specialists [41]. Long-term opioid abuse and social outcomes following opioid prescription for ALBP have not been summarized in a systematic review, which makes it challenging for clinicians to make treatment-related decisions and for patients to make informed choices. Given the addictive potential of opioid analgesics and the individual and societal consequences associated with misuse, it is important to systematically review the evidence reporting long-term outcomes associated with opioid use for ALBP.

This review will make significant contributions to prescribing practices of opioids for ALBP, a common symptom for many adults. We anticipate that this review will provide evidence-based outcomes of prescription opioids for patients with ALBP. This will make substantial contributions to healthcare practice for primary care physicians as well as specialists prescribing opioid analgesics and managing ALBP, including emergency physicians, surgeons, anaesthesiologists and other pain specialists. Health policy researchers will also benefit from the evidence in the development of evidence-based guidelines. Various knowledge users, including primary care and emergency physicians, a pharmacist and an information specialist, have been involved in the development of the research question and will continue to contribute their content and technical expertise throughout the systematic review process. This review will generate evidence-based recommendations to improve the prescribers’ and dispensers’ knowledge and education of the potential risks of opioid prescription in this population. As a result of this evidence, we expect to see changes in opioid analgesic prescribing patterns to prevent long-term adverse events related to opioid use in ALBP patients.

## Additional files

Additional file 1: The PRISMA-P checklist. (PDF 314 kb)

Additional file 2: The data extraction form. (PDF 181 kb)

## Abbreviations

ALBP: Acute lower back pain
MOOSE: Meta-analysis of Observational Studies in Epidemiology
NOS: Newcastle-Ottawa Scale
PRISMA: Preferred Reporting Items for Systematic Reviews and Meta-Analyses
RCT: Randomized controlled trial
