# Supplementary material for: Adverse outcomes associated with opioid prescription for acute low back pain: a systematic review protocol
Source: Syst Rev. 2017 Aug 14;6:163. doi: 10.1186/s13643-017-0556-x (PMC5557568; doi:10.1186/s13643-017-0556-x)
Supplement: Supplementary file 2 — The data extraction form. (PDF 181 kb) [file 13643_2017_556_MOESM2_ESM.pdf]

Appendix 1:

Data Extraction Form

Study ID: _____________ Reviewer Initials: ________

Publication Details

Author (last name, first initial): ______________________________ Year: ________________

Title: ____

Journal: ___________________________________ Country: ___________________________

Methods

Study design: _____________________ Study setting: _________________________________

Length of study: ______________________

Description of sample: ____

Definition of ALBP:____

Exposure: ________________________________ Intervention (if applicable): ______________

Demographics

Number of participants: Total: _____ Men: _____ Women: _____ Per group: _______________

Mean age (SD): Total: ___________ Men: _________ Women: __________

Per group: ____

Ethnicity:____

Outcome measurements:

Efficacy outcome

Schober test: ____

Pain measurement: ____

Oswestry disability questionnaire:

____

Modified Zung questionnaire:

____

Modified somatic perception questionnaire:

____

Adverse events outcome:

Incidence of misuse:____

Opioid withdrawal symptoms:

____

Physical adverse events: ____

Social adversity:

____

Mortality: ____

Comments:

____

Results

Statistical methods: ______________________________ Adjusted for: ___________________

Coefficient: ______________________95% CI: _______________________ p-value: _______

Findings: ____

Limitations: ____

Inclusion Criteria

RCT or observational study design examining outcome of prescription opioid use for ALBP

Participants aged 18 years or older

Exclusion Criteria

Pilot or feasibility studies

Patients with comorbid use disorder

Additional Comments:

____

____
